# Systematic Study on MIL-100(Fe) Synthesis Conditions to Enhance Its Properties as a Green Material for CO_2_ Capture

**DOI:** 10.1021/acsomega.5c03761

**Published:** 2025-07-21

**Authors:** Soňa Lisníková, Petr Novák

**Affiliations:** Department of Experimental Physics, Faculty of Science, Palacký University Olomouc, 17. listopadu 12, Olomouc CZ-77146, Czech Republic

## Abstract

This study investigates the optimization of MIL-100­(Fe) metal–organic framework (MOF) synthesis for enhanced CO_2_ adsorption, focusing on the effects of the reaction time, initial pressure, and precursor concentration on the BET surface area, crystallinity, and pore size distribution. Through hydrothermal synthesis, three MIL-100­(Fe) series were developed to examine the relationship between structural parameters and CO_2_ uptake, characterized by powder X-ray diffraction (XRD), adsorption analysis, and Mössbauer spectroscopy. The results show that higher precursor concentrations lead to increased crystallinity and surface area, with BET values reaching a peak at 1775 m^2^/g. The sample with the optimal precursor concentration demonstrated the highest CO_2_ uptake at 1.91 mmol/g, likely due to the presence of fine hematite nanoparticles within the structure. Additionally, the samples exhibited excellent stability and reusability in the cyclic CO_2_ sorption experiments. These findings provide valuable insights into the synthesis-structure–property relationships in MIL-100­(Fe), enhancing its potential for CO_2_ capture and environmental remediation.

## Graphical abstract

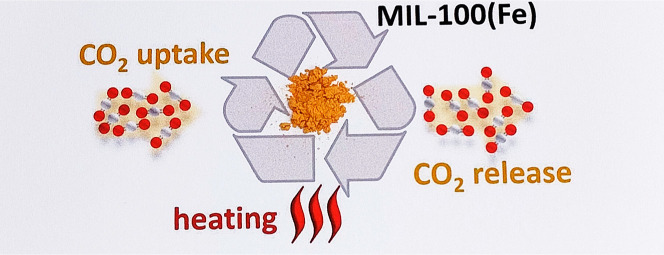

## Introduction

Global warming has emerged as a critical issue in recent years, driven by the increasing concentration of greenhouse gases in the atmosphere, particularly carbon dioxide.^1–4^ The rise in CO_2_ levels, primarily due to human activities such as burning fossil fuels and deforestation, has led to significant climate changes, including higher global temperatures, more frequent extreme weather events, CO_2_-induced ocean acidification, acidic rains, and the impact on organisms and the ecosystem.^5–7^ To address this challenge, innovative solutions for capturing and storing CO_2_ are needed, including the development of new porous materials and the optimization of their structures to enhance CO_2_ capture and storage.^8^ Among the various materials studied for CO_2_ sorption, Metal–Organic Frameworks (MOFs) have shown great promise.^9,10^

MOFs have attracted significant interest due to their exceptional characteristics, such as high surface area and ultrahigh porosity, customizable chemical structures, low density, and variable pore sizes.^11,12^ Their one- or more-dimensional structure, consisting of metal ions or clusters coordinated to organic ligands, can be precisely tuned to target specific gases, enhancing their efficiency in CO_2_ capture and storage.^13–17^ This tunability allows MOFs to adapt to different environmental conditions, positioning them as a leading material in carbon capture and storage technologies aimed at mitigating climate change.^6,18,19^

From the perspective of a wide range of practical applications, properties such as thermal and chemical stability are essential for these materials. Among the many MOFs developed, MIL-100­(Fe) (MIL stands for Material from Institut Lavoisier) is particularly notable for exhibiting these characteristics. It is composed of iron­(III) clusters and trimesic acid (benzene-1,3,5-tricarboxylate) ligands. The chemical structure forms a three-dimensional porous framework with two types of large mesoporous cages (25 and 29 Å) and two types of microporous windows, pentagonal and hexagonal (5–9 Å), which contribute to its remarkably high surface area and pore volume.^20,21^ The advantage of this material also lies in its exceptional stability under humid conditions.^22–24^ These properties make MIL-100­(Fe) relevant in fields such as gas storage and separation, where its high surface area enables the efficient adsorption of gases such as CO_2_, CH_4_, and H_2_.

Teerachawanwong et al.^25^ investigated the CO_2_ adsorption capacity of MIL-100­(Cr, Fe), reporting adsorption values ranging from 0.86 to 1.89 mmol/g at a pressure of 1 bar. Their findings indicated that samples with higher crystallinity, which also exhibited greater specific surface area, demonstrated superior adsorption performance. Similar CO_2_ sorption results for MIL-100­(Fe) have been documented by Le et al.,^26^ who also highlighted the material’s potential for reusable application as demonstrated through cyclic CO_2_ sorption testing. Existing studies have focused on enhancing the CO_2_ sorption capacity through the further functionalization of MIL-100­(Fe). Mei et al.^27^ reported an increase in CO_2_ capacity and selectivity for DOBDC-functionalized MIL-100­(Fe) compared to pure MIL-100­(Fe). Additionally, functionalization with amino groups has been identified as a promising approach for enhancing CO_2_ sorption capacity in other MOF structures.^28,29^ However, Steenhaut et al.^30^ observed a decrease in CO_2_ capacity for MIL-100 materials when functionalized with amino groups.

Additionally, MIL-100­(Fe) has shown promise in catalysis due to the accessible iron sites within its structure, which can act as active centers for various catalytic reactions.^31^ Its biocompatibility and low toxicity also open up potential applications in biomedicine, such as drug delivery and imaging.^32,33^ The versatility and robust performance of MIL-100­(Fe) in these diverse applications underscore its importance and drive continued research into optimizing its synthesis and properties for enhanced functionality.

Recent studies have explored various approaches to the synthesis of MIL-100­(Fe), including dry-gel conversion,^34,35^ low-temperature methods,^36,37^ green mechanochemical process, and solvent-free synthesis.^38–40^ However, MIL-100­(Fe) is most commonly prepared through solvothermal or hydrothermal techniques, as these methods allow for precise control over critical product properties, such as specific surface area, porosity, and crystallinity. The synthesis typically involves mixing an iron precursor with H_3_BTC in deionized water or another solvent, sometimes using auxiliary reagents such as mineralizing agents (e.g., HF). The reaction proceeds at elevated temperatures and pressures within an autoclave.^41^

To achieve the desired properties of the resulting nanomaterial, it is crucial to optimize key synthesis parameters, including the iron precursor, reaction time, temperature, the use of mineralizing agents, etc., and to assess the impact of these conditions on MIL-100­(Fe). Seo et al.^42^ investigated the effects of various iron precursors, the presence or absence of hydrofluoric acid (HF), and the concentration of the precursor on specific surface area, total pore volume, and product yield. Similarly, Han et al.^38^ conducted solvent-free syntheses while examining these factors. Mineralizing agents, in particular, are believed to enhance the crystallinity of the final product. However, given the toxicity and environmental concerns associated with the commonly used mineralizer HF, Fang et al.^43^ compared MIL-100­(Fe) synthesized without a mineralizer to those produced with HF, TMAOH, and Na_2_CO_3_.

In this study, we present an optimized method for producing highly crystalline MIL-100­(Fe) without the use of a mineralizing agent. We also systematically investigate the influence of the reaction time, initial pressure, and precursor concentration on the BET surface area, crystallinity, and pore size distribution of synthesized MIL-100­(Fe). The pore size distribution was determined using the modern NLDFT (nonlocal density functional theory) method, which is particularly well-suited for materials with complex pore structures, where pore dimensions span the micro- and mesoporous regions. Furthermore, the CO_2_ adsorption capacity of the prepared materials was thoroughly evaluated, including comprehensive cyclic sorption experiments to assess their durability and potential for repeated use. These findings highlight the material’s potential in CO_2_ capture applications, with a focus on its long-term performance and stability under varying experimental conditions.

## Experimental Section

### Chemicals

Iron­(III) nitrate nonahydrate (Fe­(NO_3_)_3_·9H_2_0) was purchased from Penta Ltd. 1,3,5-benzentricarboxylic acid (H_3_BTC, 98%) was obtained from Thermo Fisher Scientific Inc. Both reagents were used without further purification.

#### Synthesis of Three MIL-100­(Fe) Sample Series

Three series of MIL-100­(Fe) samples were synthesized by using the hydrothermal method (Table 1). Deionized water, previously purged with nitrogen to remove any dissolved oxygen, was used as the solvent. In each synthesis, 9 mmol of iron nitrate was dissolved in prepared water (z mL), followed by the addition of 6 mmol of benzenetricarboxylic acid. The precursor mixture was then stirred for 1 h at room temperature (600 rpm) to homogenize the solution.

Subsequently, the mixture was transferred into a PTFE-lined stainless-steel autoclave. After evacuation and pressurization with nitrogen gas to an initial pressure (*y* bar), the autoclave was heated to 160 °C for a specified duration (*x* hours). Magnetic stirring during the synthesis ensured the homogeneity of the final product. The resulting light orange precipitate was thoroughly washed with deionized water and ethanol to remove impurities, followed by centrifugation to separate the solid MOF from the liquid phase.

**Table 1:** Reaction Conditions for Three MIL-100­(Fe) Series

Reaction time series:	
Reaction time	*x* = 1, 2, 4, 6, 8, and 12 h
Initial pressure	*y* = 1 bar
Amount of DI water	*z* = 18 mL
Ratio precursors [mmol]/DI water [mL]	0.83:1

Initial pressure series:	
Reaction time	*x* = 2 h
Initial pressure	*y* = 0, 0.5, 1, 4.75, and 9 bar
Amount of DI water	*z* = 18 mL
Ratio precursors [mmol]/DI water [mL]	0.83:1

Precursor concentration series:	
Reaction time	*x* = 2 h
Initial pressure	*y* = 1 bar
Amount of DI water	*z* = 2.25, 4.5, 9, 18, and 36 mL
Ratio precursors [mmol]/DI water [mL]	6.66:1, 3.33:1, 1.66:1, 0.83:1 and 0.42:1

#### Autoclave

For all syntheses, a Miniclave steel (Büchiglasuster) with a volume of 300 mL, equipped with a PTFE insert and a magnetic stirrer sealed in PTFE, was used at a stirrer speed of approximately 500 rpm. The temperature of the reaction mixture was monitored by using a Pt 100 thermometer protected by a Hastelloy C22 sheath, which was placed directly in the reaction mixture. The temperature profile is shown in Figure S1 in the Supporting Information.

### Characterization Techniques

The X-ray powder diffraction patterns were collected in the 2θ range from 6° to 100° with a scan step of 0.02°. The measurements were conducted under ambient conditions using a D8 ADVANCE diffractometer (Bruker) equipped with Bragg–Brentano geometry, a LYNXEYE position-sensitive detector, and a Co tube (λ_Co_ = 1.7889 Å). The 0.6 mm divergence slit and 2.5° axial Soller slits were inserted to the primary beam path. The secondary beam path included an Fe Kβ filter and 2.5° axial Soller slits.

The morphology of powdered samples was monitored using a scanning electron microscope, VEGA3 LMU, equipped with an Everhart-Thornley-type secondary electron detector (Tescan). The accelerating voltage was set to 30 kV. SEM observations were made after depositing a 20 nm Cu layer on the samples using a sputtering device, Q 150T ES (Quorum Technologies), to increase the conductivity.

The N_2_ adsorption–desorption isotherms at 77 K for prepared MIL-100­(Fe) samples were measured using the Autosorb iQ adsorption analyzer from Quantachrome Instruments Anton Paar, which uses the static volumetric technique. Prior to measurement, each sample was outgassed for 12 h at 150 °C and subsequently for 12 h at room temperature. The BET areas of the prepared samples were determined using the multipoint Brunauer–Emmett–Teller (BET) model, with the range of BET adsorption points established following Rouquerol’s criteria.^44^

The Ar adsorption–desorption isotherms at 87 K were collected on the same analyzer equipped with a Cryosync. Prior to measurement, each sample was outgassed for 12 h at 150 °C and subsequently for 12 h at room temperature. The pore volume, pore width, and pore size distribution were derived from the Ar adsorption–desorption isotherms using a nonlocal density functional theory (NLDFT) model in the Autosorb software.

The CO_2_ sorption experiments on MIL-100­(Fe) samples were performed by using an Autosorb iQ apparatus with an external Julabo bath. All CO_2_ adsorption measurements were carried out up to a pressure of 1 bar. Prior to measurement, the samples were degassed for 12 h at 250 °C, followed by an additional 12 h at room temperature. Comparative measurements of various samples were executed at 25 °C. During cyclic measurements, each sample was redegassed for 12 h at 250 °C and 12 h at room temperature before subsequent measurements.

Transmission ^57^Fe Mössbauer spectroscopy was conducted at room temperature using the OLTWINS dual spectrometer developed at Palacký University Olomouc, Czech Republic. This spectrometer is based on electronics^45^ and utilizes a drive unit,^46^ and the movement is controlled using an autotuning procedure.^47^ For the low-temperature measurement, the sample was placed in a cryogen-free, closed-cycle cryogenic system (Cryostation, Montana Instruments). The radioactive source ^57^Co, embedded in a rhodium matrix (RITVERC), was employed, and the recorded transmission spectra were analyzed using MossWinn 4.0 software.^48^ The velocity axis calibration was performed by measuring the spectrum of metallic α-iron at room temperature.

## Results and Discussion

### Sample Preparation and Synthesis Conditions

Three series of MIL-100­(Fe) samples were prepared in order to investigate the extent of CO_2_ uptake within this structure depending on the BET surface area, crystallinity, and pore size distribution. Additionally, this study aims to elucidate how these parameters are influenced by initial conditions, including reaction time, initial pressure, and precursor concentration. The samples are named according to the varying parameter of each series; for example, in the reaction time series, the samples were labeled as follows: MIL-100­(Fe)_1h, MIL-100­(Fe)_4h, ... Only the MIL-100­(Fe)_*0.83:1_2h_1 bar* sample bears a specialized designation, signifying its inclusion within each of the three experimental series. All details regarding the synthesis and the chosen values of individual parameters can be found in the Experimental Section.

All syntheses were conducted by using a one-pot method within an autoclave under an inert nitrogen atmosphere. Moreover, during the reaction, the actual pressure inside the autoclave was monitored. The highest values achieved for each sample are depicted in the graph (see Figure 1). The absolute highest pressure was attained through utilization of a high input pressure. Furthermore, it becomes evident that not only the input pressure but also the precursor concentration significantly influenced the resulting pressure achieved. Subsequent measurements indicate that the most favorable results were achieved when the initial pressures ranged from 0.5 to 1 bar.

**Figure 1:**
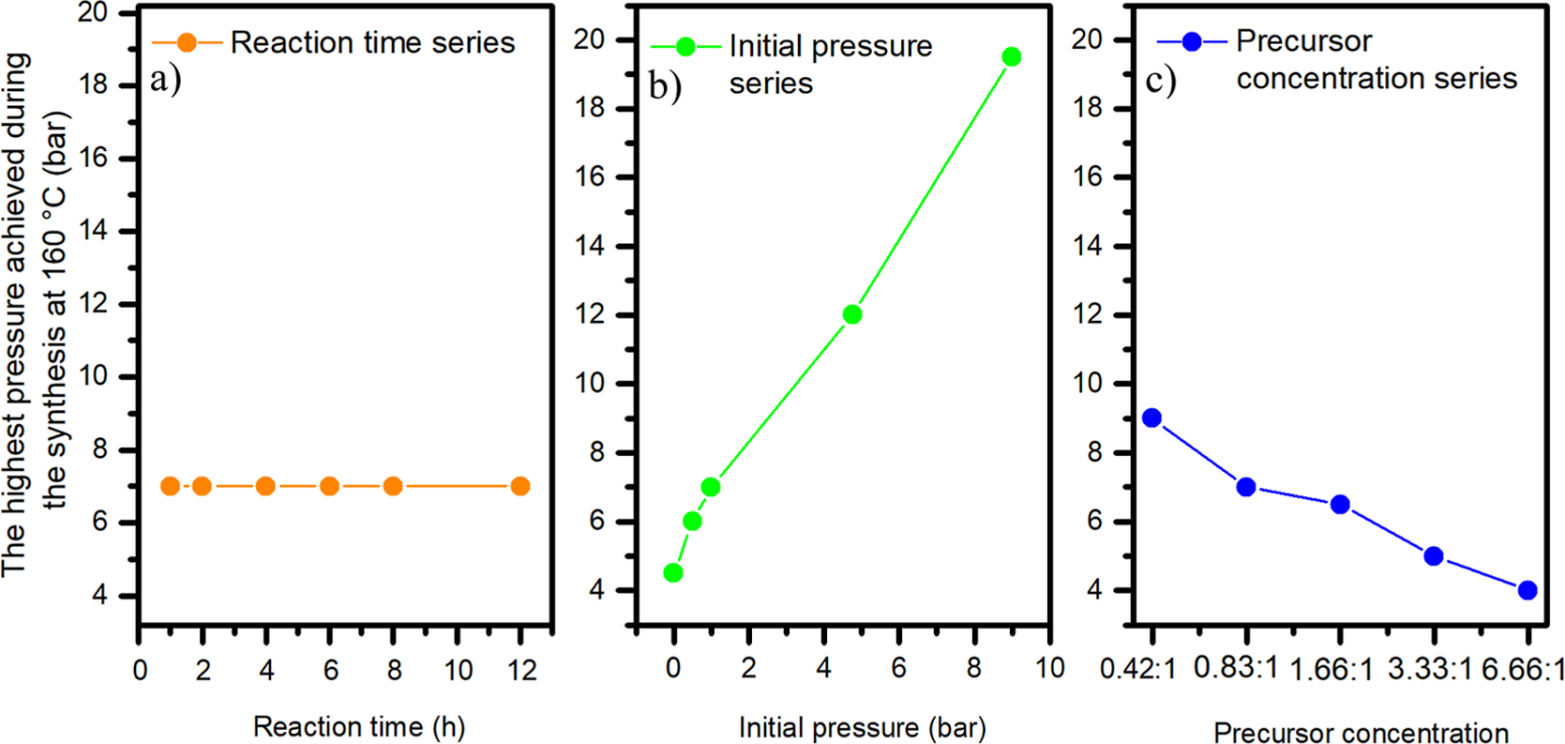
Development of maximum pressure during synthesis depending on input parameters: (a) reaction time, (b) initial pressure, and (c) precursor concentration.

### X-ray Diffraction Analysis

The crystallinity and phase purity of the MIL-100­(Fe) samples were examined by using powder X-ray diffraction (XRD) analysis. The XRD patterns of almost all as-synthesized MIL-100­(Fe) samples showed good agreement with the calculated pattern, indicating successful synthesis of the desired framework. Figure 2 compares the XRD patterns of samples from the precursor concentration series. Among these samples, only the MIL-100­(Fe)_0.42:1 sample contains a small percentage of hematite identifiable by XRD (see the XRD pattern in full range in Supporting Information Figure S2). All other samples from this series, as well as the other two series, are pure MIL-100­(Fe) without additional impurities. XRD patterns of reaction time series samples and initial pressure series samples are provided in Supporting Information Figures S3 and S4.

**Figure 2:**
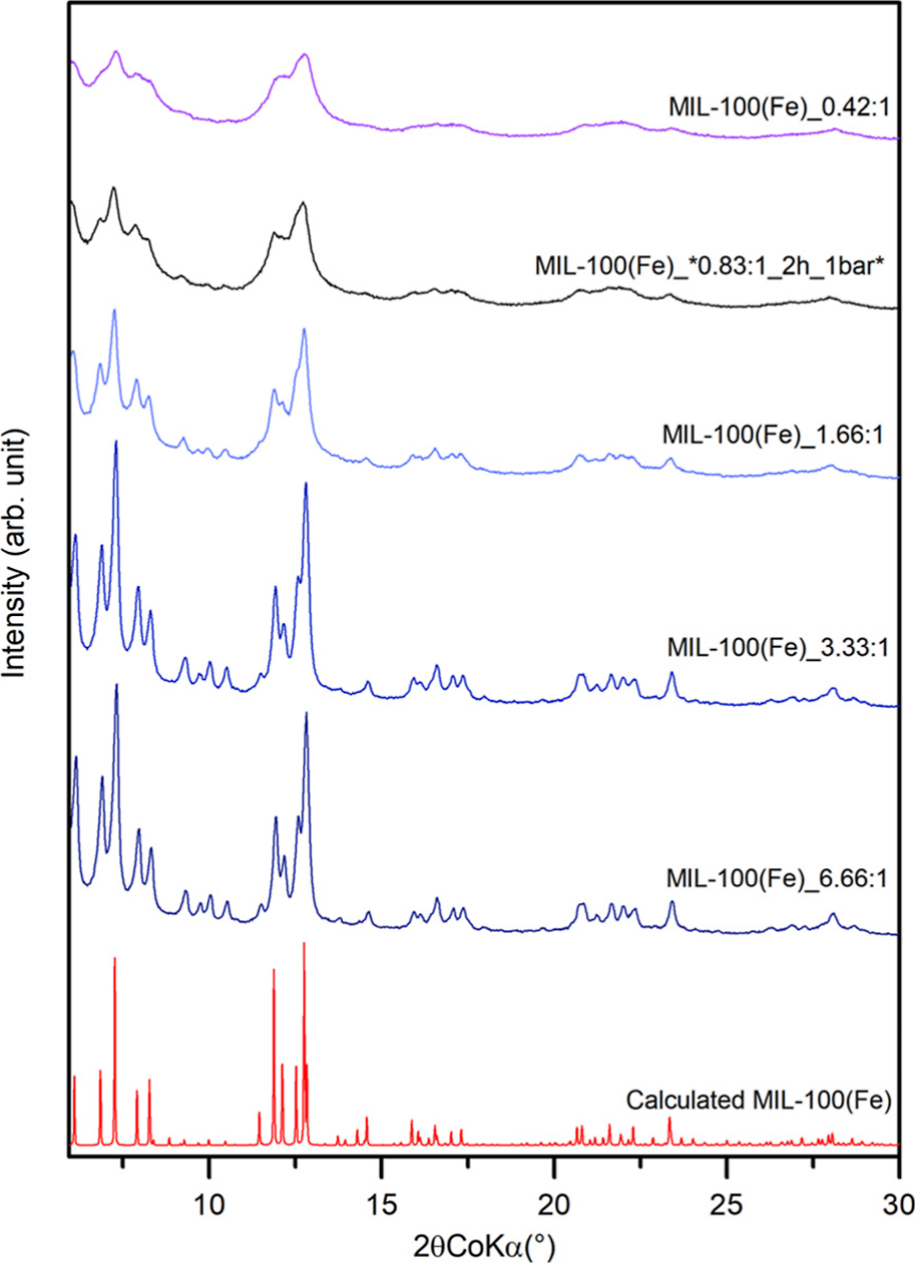
Evolution of the sample crystallinity through the precursor concentration series.

The XRD patterns in Figure 2 demonstrate how the crystallinity of the prepared MIL-100­(Fe) samples changes across a precursor concentration series. As the concentration of the initial solution increases, the crystallinity of the resulting sample also improves. However, extending the reaction time or altering the initial pressure did not exhibit such a trend.

### BET Surface Area

Second, the change in the BET surface area of MIL-100­(Fe) samples was investigated, and the parameters leading to optimal results were identified. Figure 3 illustrates the evolution of the BET surface area across all three series. Notably, the highest BET surface area was achieved within the concentration series, specifically for the MIL-100­(Fe)_3.33:1 sample, which exhibited a remarkable BET surface area of 1775 m^2^/g. In contrast, the smallest BET surface area (1247 m^2^/g) was observed for the MIL-100­(Fe)_1h sample from the reaction time series. In Figure 3, the BET surface area displayed a “wave-shaped” trend as a function of reaction time. This behavior could be explained by a combination of crystallization, defect formation, and structural changes. During the initial stages (1–4 h), rapid nucleation results in small, highly porous MIL-100­(Fe) particles, leading to a high BET surface area. As the reaction progresses (4–6 h), Ostwald ripening occurs, where smaller crystallites dissolve and redeposit onto larger ones, leading to densification and a decrease in the surface area. At longer reaction times (6–12 h), the crystallites stabilize, the framework reorganizes, and porosity increases again, restoring or even enhancing the BET surface area.^49^ Overall, the development of the BET surface area of MIL-100­(Fe) is not straightforward; it can be tuned by adjusting the synthesis parameters and its specific combination. The values of the BET surface areas of all measured samples can be found in Table S1.

**Figure 3:**
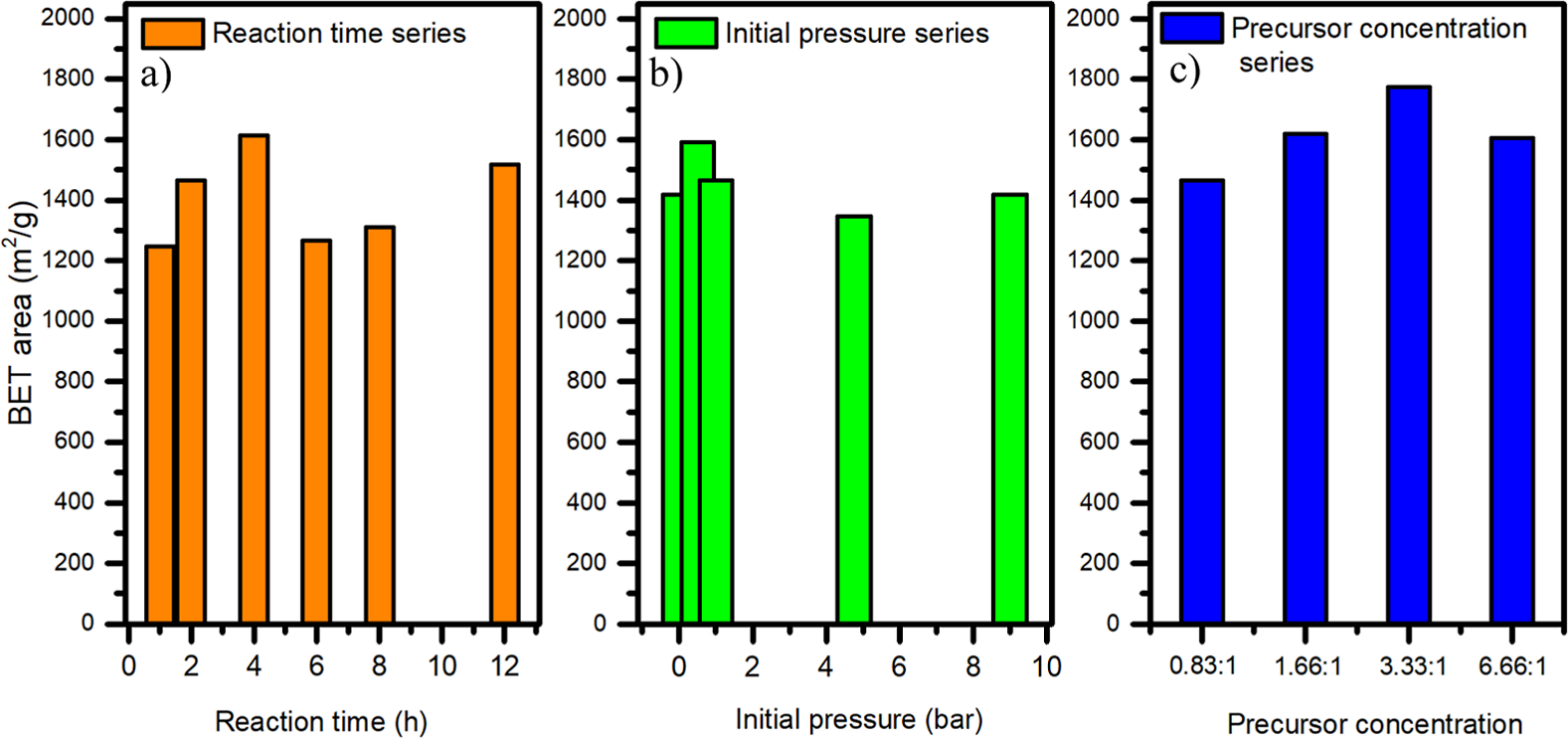
Variation of the BET surface area of MIL-100­(Fe) with respect to changing (a) reaction time, (b) initial pressure, and (c) precursor concentration.

The N_2_ adsorption–desorption isotherms of the concentration series samples are shown in Figure 4, while those for the other two series are presented in Supporting Information Figure S5. All samples exhibit an isotherm of type I­(b), characteristic of microporous materials with a pore size distribution spanning a broader range, including narrow mesopores (<∼2.5 nm). Enhanced uptake at low relative pressure is associated with micropore filling. Additionally, certain isotherms exhibit a small, narrow hysteresis loop. Notably, the isotherm of the MIL-100­(Fe)_1.66:1 sample shows a slightly more pronounced hysteresis loop, identified as a type H4 loop according to IUPAC classification. H4 loops are commonly found in some micromesoporous carbons.^50^

**Figure 4:**
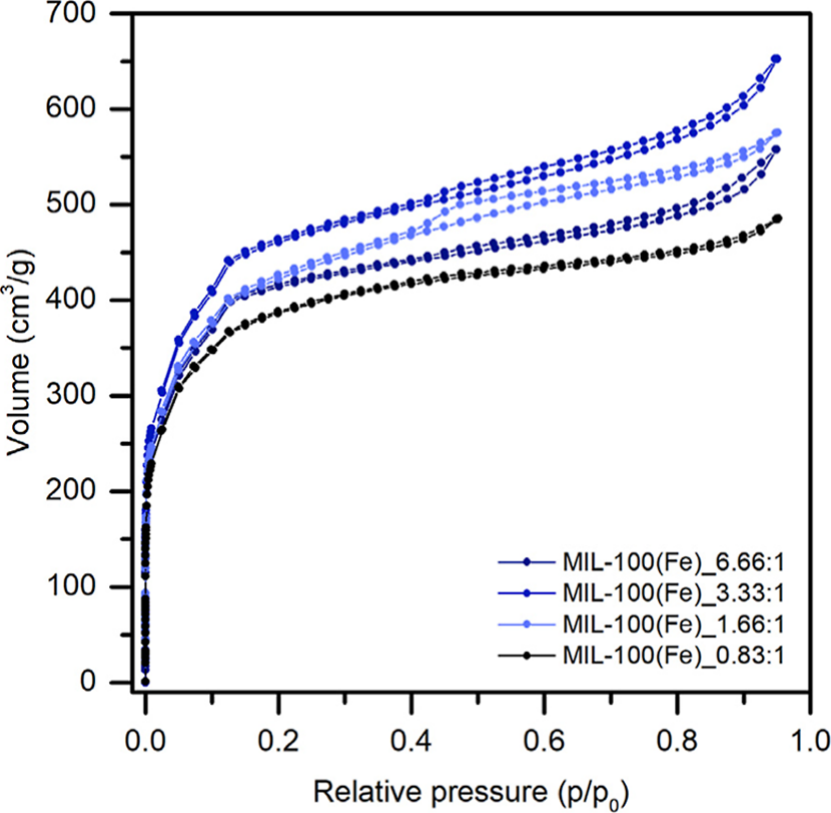
N_2_ adsorption–desorption isotherms of the concentration series samples.

#### ^57^Fe Mössbauer Analysis

The results of the Mössbauer spectral measurements for the selected samples are shown in Figure 5, with one of them depicted in Figure 6. The selected samples comprised four from the precursor concentration series, including the sample with the highest crystallinity (MIL-100­(Fe)_6.66:1) and the one with the largest surface area (MIL-100­(Fe)_3.33:1). Additionally, two samples were chosen from the reaction time series: one representing the worst crystalline sample (MIL-100­(Fe)_4h) and the other exhibiting the lowest surface area (MIL-100­(Fe)_1h) in comparison to samples across all series. As previously published, the MIL_100­(Fe) material appears in the Mössbauer spectrum as an asymmetric doublet.^51^ From Figure 5, it is evident that this asymmetry varies for different samples (the results of Mössbauer spectrometry measurements for all samples are provided in Figure S6, Table S3 and Table S4). This asymmetry can be evaluated using a range of doublet components.^21^ For the evaluation of almost all Mössbauer spectra, a single component was utilized, specifically an asymmetric doublet. When the evaluation of this asymmetry is shown in Figure 5, it is illustrated in the graph as the ratio of the first L_1_ (left) and second L_2_ (right) spectral lines. The trend of the asymmetry ratio of spectral lines is consistent with the N_2_ results, except for samples MIL-100­(Fe)_4h and MIL-100­(Fe)_*0.83:1_2h_1 bar*, which are swapped, possibly due to measurement errors. It holds that the greater the asymmetry, the larger the BET area.

**Figure 5:**
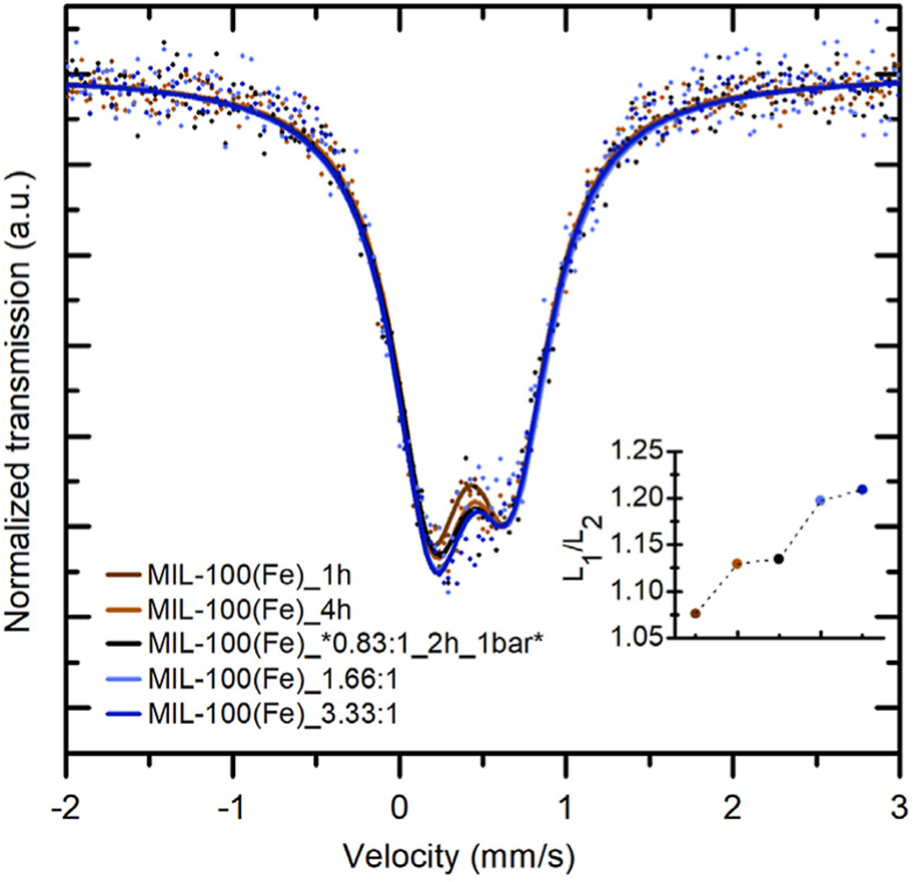
Comparison of the Mössbauer spectra of selected samples.

**Figure 6:**
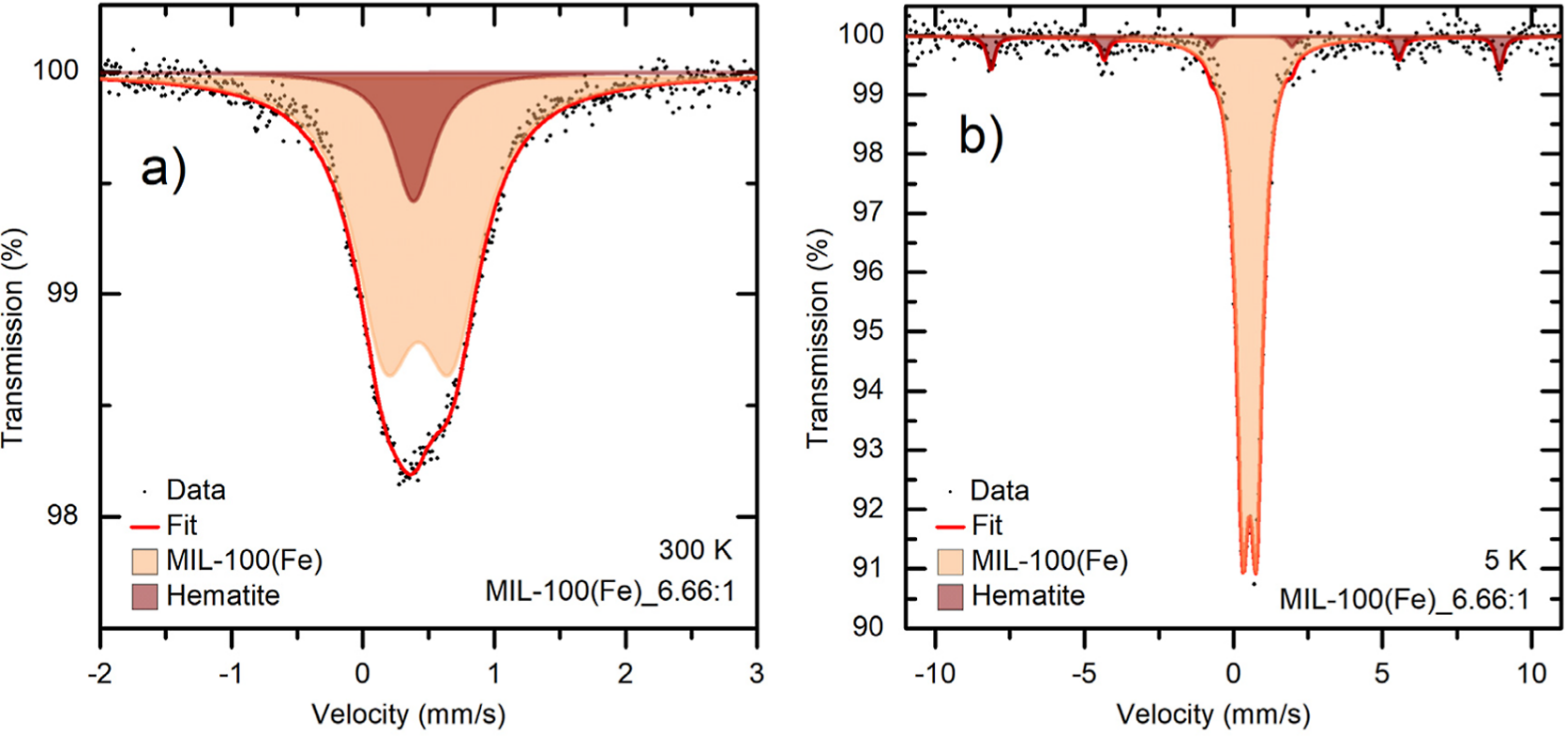
Mössbauer spectrum of the MIL-100­(Fe)_6.66:1 sample measured at (a) room temperature and at (b) 5 K.

The MIL-100­(Fe)_6.66:1 sample could not be evaluated using an asymmetric doublet because it was the only selected sample that contained an additional spectral component, as shown in Figure 6a). Low-temperature Mössbauer spectral measurements (5 K) revealed that this component is hematite, as shown in Figure 6b). At room temperature, it is in a superparamagnetic state because it consists of very small particles, and it appears as a singlet component in the spectrum. All hyperfine parameters are listed in Table S4.

### Porous Properties and CO_2_ Sorption Capacity

In addition to N_2_ adsorption measurements, Ar adsorption–desorption isotherms were also conducted to characterize the porous properties of the MIL-100­(Fe) samples. Argon adsorption at 87 K is particularly suitable for this analysis due to its inert, monatomic nature, which avoids specific interactions with surface functional groups.^52,53^ From the Ar isotherms, the total pore volume and pore size distribution for the selected samples were subsequently calculated. The NLDFT method was employed for pore size distribution analysis, as it has been shown to provide more accurate results compared to classical methods.^52,53^ These results are summarized in Table 2, alongside the specific surface area determined from Ar measurements.

**Table 2:** BET and Ar Areas, Pore Volume, Pore Width, and CO_2_ Sorption of Selected MIL-100­(Fe) Samples

sample	BET area [m^2^/g]	Ar area [m^2^/g]	pore volume [cm^3^/g]	pore width [nm]	CO_2_ sorption at 298 K and 1 bar [mmol/g]
MIL-100(Fe)_1h	1247	1909	0.548	1.946	1.66
MIL-100(Fe)_4h	1615	2048	0.680	1.946	1.76
MIL-100(Fe)_*0.83:1_2h_1 bar*	1466	1977	0.659	1.946	1.56
MIL-100(Fe)_1.66:1	1620	1960	0.768	2.397	1.77
MIL-100(Fe)_3.33:1	1775	2135	0.911	2.397	1.48
MIL-100(Fe)_6.66:1	1607	2060	0.779	2.397	1.91

Notably, it was found that changes in sample crystallinity, as observed through X-ray diffraction measurements (Figure 2), were not directly correlated with the BET/Ar surface area. Instead, they were more closely associated with variations in the pore size distribution.

The improvement in crystallinity appeared to be linked to changes in the pore width. Specifically, more crystalline samples exhibited a pore width of approximately 2.397 nm, while less crystalline samples possessed a narrower pore width of 1.946 nm. These values corresponded to the highly porous three-dimensional structure of MIL-100­(Fe), which consisted of two distinct types of mesoporous cages. Interestingly, the experimentally determined pore widths were slightly underestimated compared with theoretically calculated values (2.5 and 2.9 nm). These mesoporous cages were accessible through pentagonal and hexagonal windows with free diameters of approximately 0.55 and 0.86 nm, respectively.^20,21^ These features were also evident in the individual plots presented in Figure 7. Notably, the MIL-100­(Fe)_1.66:1 sample exhibited a more pronounced representation of larger pores around 4.25 nm, which may be related to the distinct hysteresis loop observed in the BET isotherm (Figure 4).

**Figure 7:**
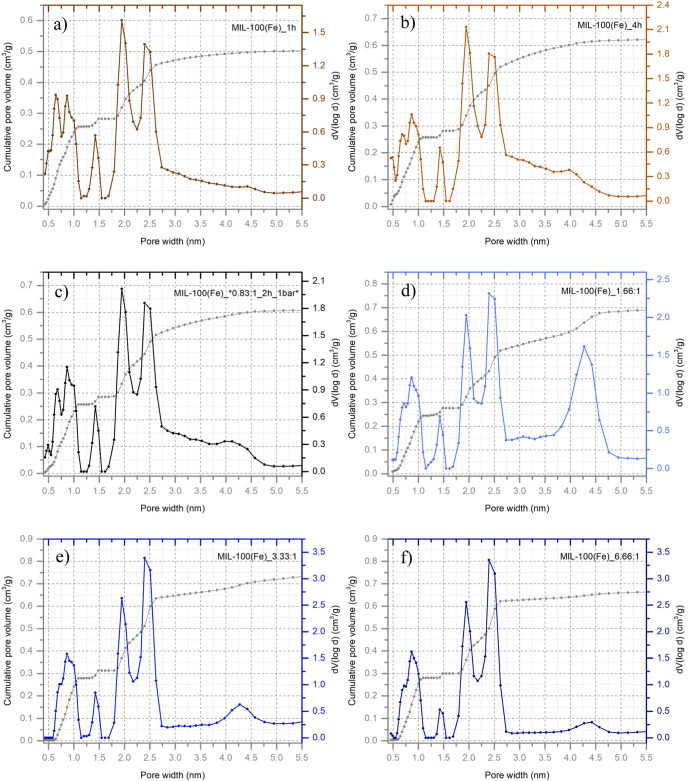
NLDFT pore size distribution plots with cumulative pore volume of selected MIL-100­(Fe) samples: (a) MIL-100­(Fe)_1h, (b) MIL-100­(Fe)_4h, (c) MIL-100­(Fe)_*0.83:1_2h_1 bar*, (d) MIL-100­(Fe)_1.66:1, (e) MIL-100­(Fe)_3.33:1, and (f) MIL-100­(Fe)_6.66:1.

The CO_2_ sorption capacity of various MIL-100­(Fe) samples was systematically evaluated. Figure 8a presents the CO_2_ adsorption isotherms measured at 298 K for the same samples whose porosity had been previously assessed. The measured values do not exhibit a clear trend regarding CO_2_ uptake in relation to pore size and total pore volume, which may be attributed to the inability to detect pores smaller than 0.4 nm, considering that the length of a carbon dioxide molecule is approximately 0.33 nm.^54^ Among the samples analyzed, the MIL-100­(Fe)_6.66:1 sample demonstrated the highest CO_2_ uptake, achieving a value of 1.91 mmol/g. This value is twice as high as the uptake reported by Teerachawanwong et al.^25^ and 1.5 times greater than the measurements by Le et al.^26^ Additionally, Takawane et al.^55^ also reported a lower CO_2_ uptake of 0.81 mmol/g for MIL-100­(Fe) measured at 300 K. This enhanced CO_2_ sorption capacity could be associated with the presence of a very small quantity of extremely fine hematite nanoparticles embedded within the MOF structure, as revealed by low-temperature Mössbauer spectroscopy. All measured CO_2_ uptake values for the individual samples are detailed in Table 2.

**Figure 8:**
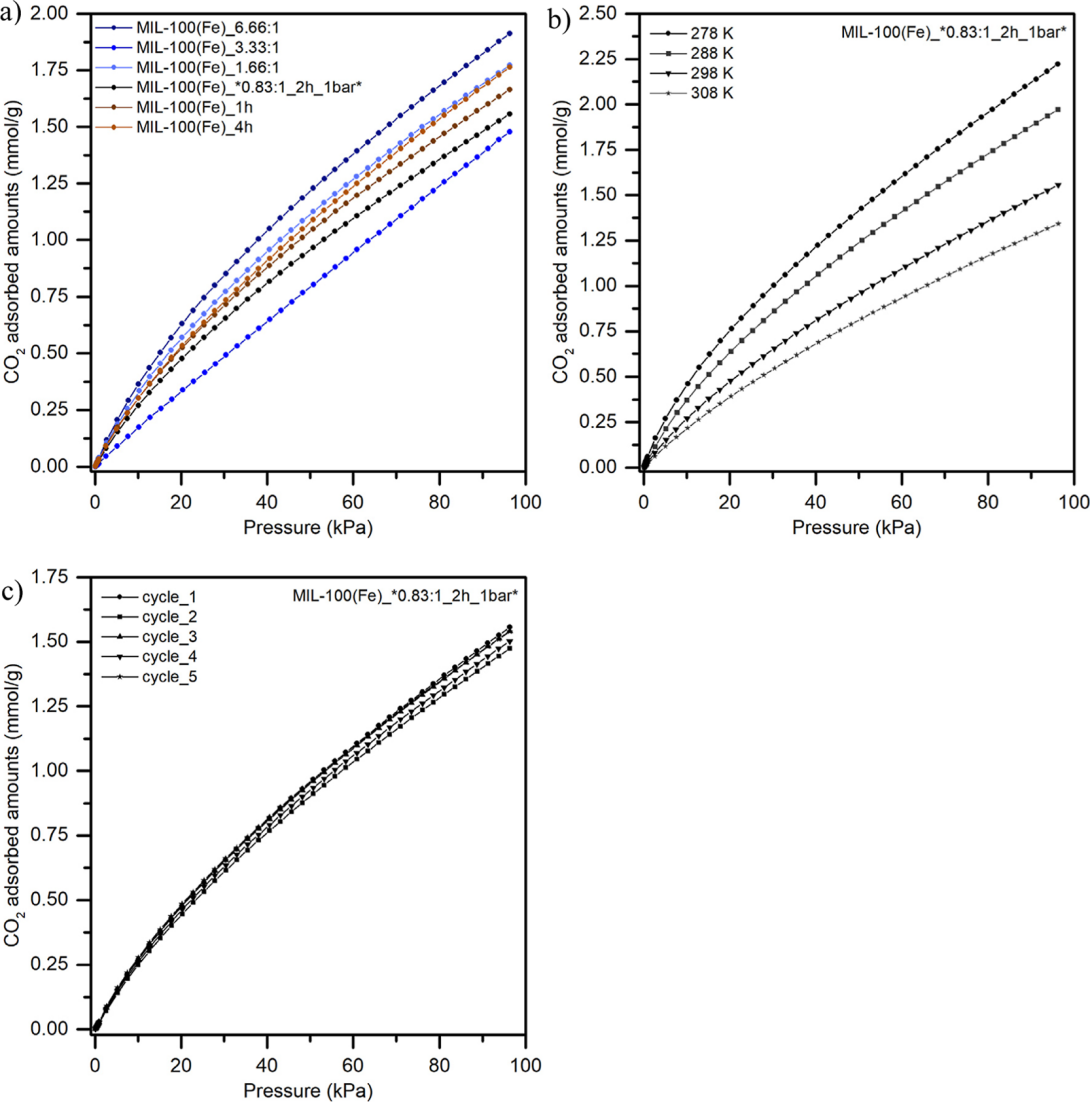
CO_2_ adsorption isotherms of (a) selected MIL-100­(Fe) samples at 298 K, (b) MIL-100­(Fe)_*0.83:1_2h_1 bar* sample at different temperatures, and (c) MIL-100­(Fe)_*0.83:1_2h_1 bar* sample for five cycles at 298 K.

The temperature dependence of the CO_2_ sorption capacity of MIL-100­(Fe)_*0.83:1_2h_1 bar* was evaluated over a range of 5 to 35 °C (refer to Figure 8b and Table S2). The results demonstrated a decline in CO_2_ uptake as the temperature increased, indicating that the adsorption of CO_2_ on MIL-100­(Fe)_*0.83:1_2h_1 bar* is primarily governed by physisorption, consistent with previous findings of Mei et al.^27^ This reliance on weak physical interactions between CO_2_ and the material enhances the potential for repeated use, thus extending the material’s functional lifespan. To further support this claim, cyclic CO_2_ sorption measurements were performed on MIL-100­(Fe)_*0.83:1_2h_1 bar* at 25 °C over five cycles (see Figure 8c). These measurements revealed minimal reduction in adsorption capacity between the first and fifth cycles, confirming the material’s robustness for multiple uses.

## Conclusion

A systematic study of the synthesis of MIL-100­(Fe) was conducted to optimize its properties, focusing on reaction time, initial pressure, and precursor concentration. The resulting samples exhibited varying degrees of crystallinity, high BET surface areas, and well-tuned pore sizes. While adjustments to reaction time and initial pressure did not significantly enhance crystallinity, changes in precursor concentration had a more pronounced effect. The crystallinity of the material improved with increasing precursor concentration, and the highest BET surface area of 1775 m^2^/g was achieved at a precursor concentration of 3.33:1, while a CO_2_ uptake of 1.91 mmol/g was observed for the sample prepared at 6.66:1. The enhancement in the CO_2_ adsorption capacity could be attributed to the finely dispersed hematite nanoparticles within the MOF framework, as confirmed by low-temperature Mössbauer spectroscopy. Notably, selecting an appropriate synthesis duration of 2–4 h allows for a high surface area, eliminating the need for extended reaction times (16+ hours) reported in previous studies, thus reducing production costs. Additionally, the material demonstrated stability and reusability in cyclic adsorption tests, reinforcing its potential for sustainable CO_2_ capture applications. Given the presence of hematite nanoparticles in one of the samples, it would be valuable to further investigate their specific influence on the structural and adsorption properties of MIL-100­(Fe).

## Supplementary Material



## Notes

Complete XRD patterns, SEM images, additional N_2_ adsorption–desorption isotherms, and Mössbauer data (PDF)
