# Supplementary material for: Systematic Study on MIL-100(Fe) Synthesis Conditions to Enhance Its Properties as a Green Material for CO2 Capture
Source: ACS Omega. 2025 Jul 21;10(30):33461–70. doi: 10.1021/acsomega.5c03761 (PMC12332548; doi:10.1021/acsomega.5c03761)
Supplement: Supplementary file 1 [file ao5c03761_si_001.pdf]

Supporting Information

Systematic Study on MIL-100(Fe) Synthesis Conditions to Enhance its Properties as a Green Material for CO_2 Capture

Soňa Lisníková [1], Petr Novák [1, *]

[1] Department of Experimental Physics, Faculty of Science, Palacký University Olomouc, 17. listopadu 12, CZ-77146 Olomouc, Czech Republic
* Correspondence: petr.novak@upol.cz; Tel.: + 420 585634152

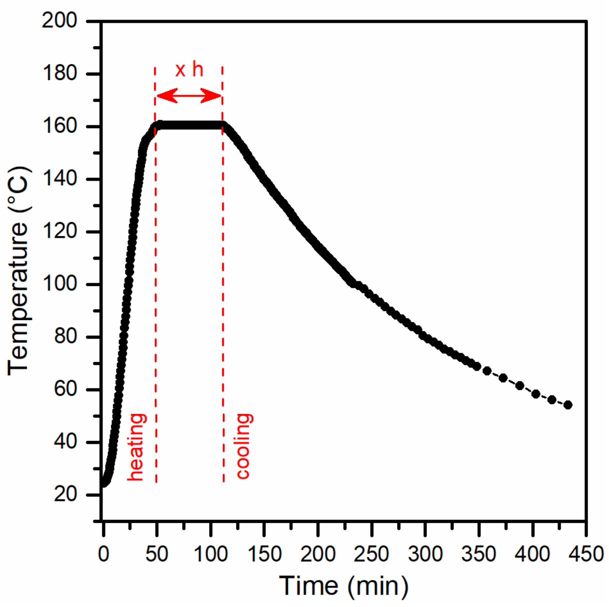

Figure S1. The temperature profile consisting of an initial heating phase, a synthesis period of x hours, and a cooling phase within the autoclave.

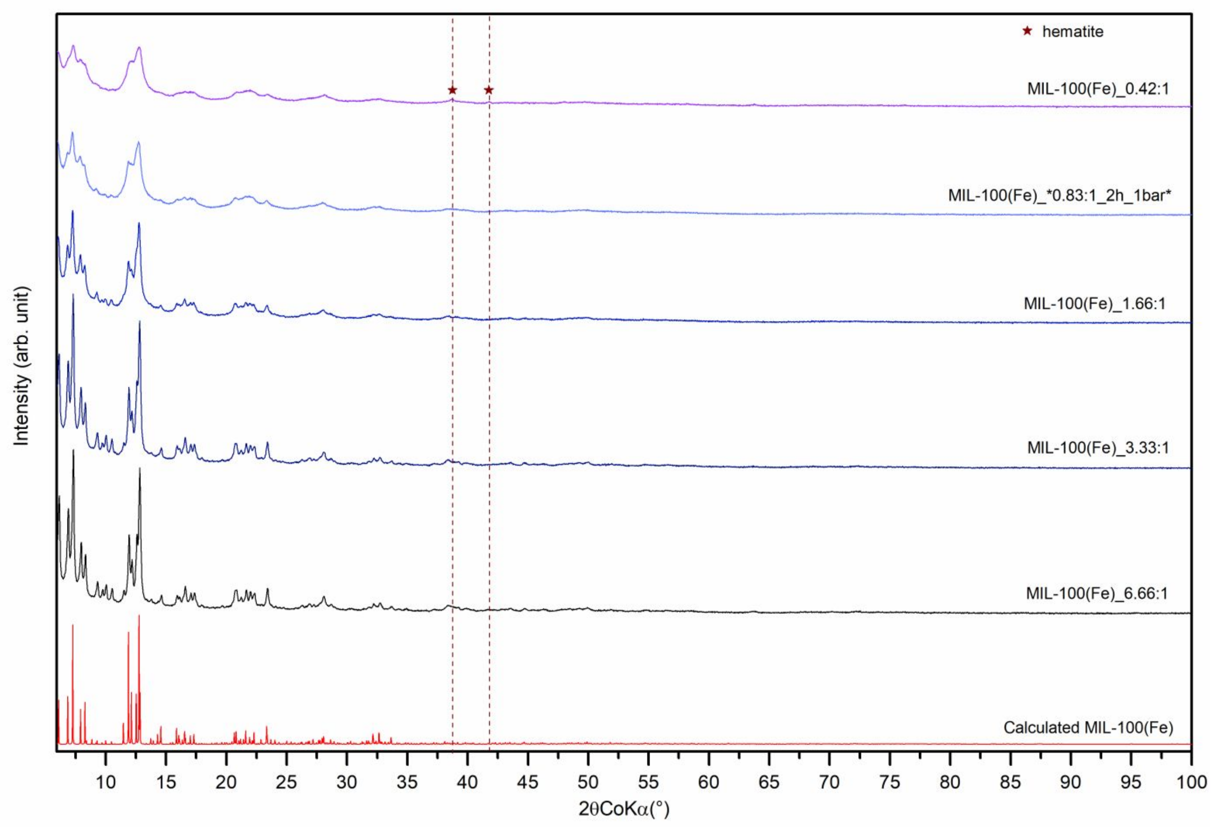

Figure S2. The evolution of the sample crystallinity through the precursor concentration series.

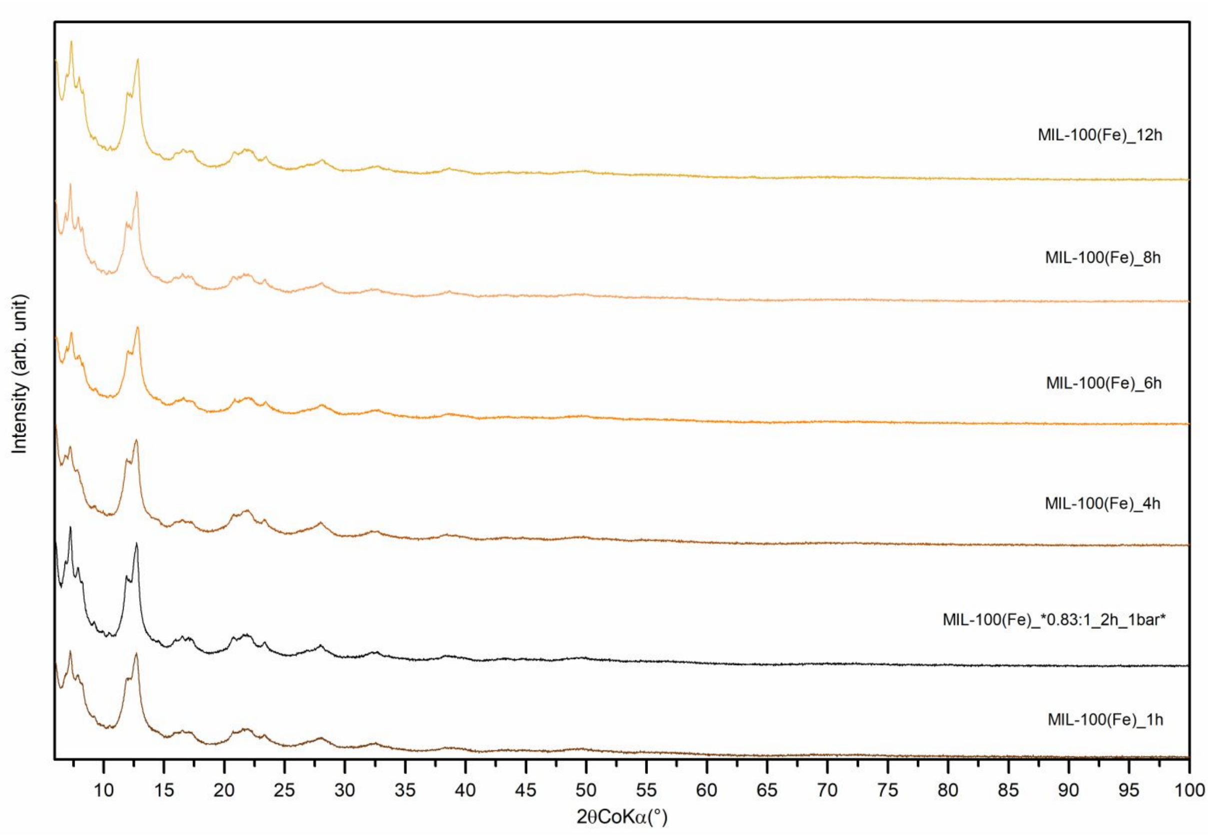

Figure S3. The evolution of the sample crystallinity through the reaction time series.

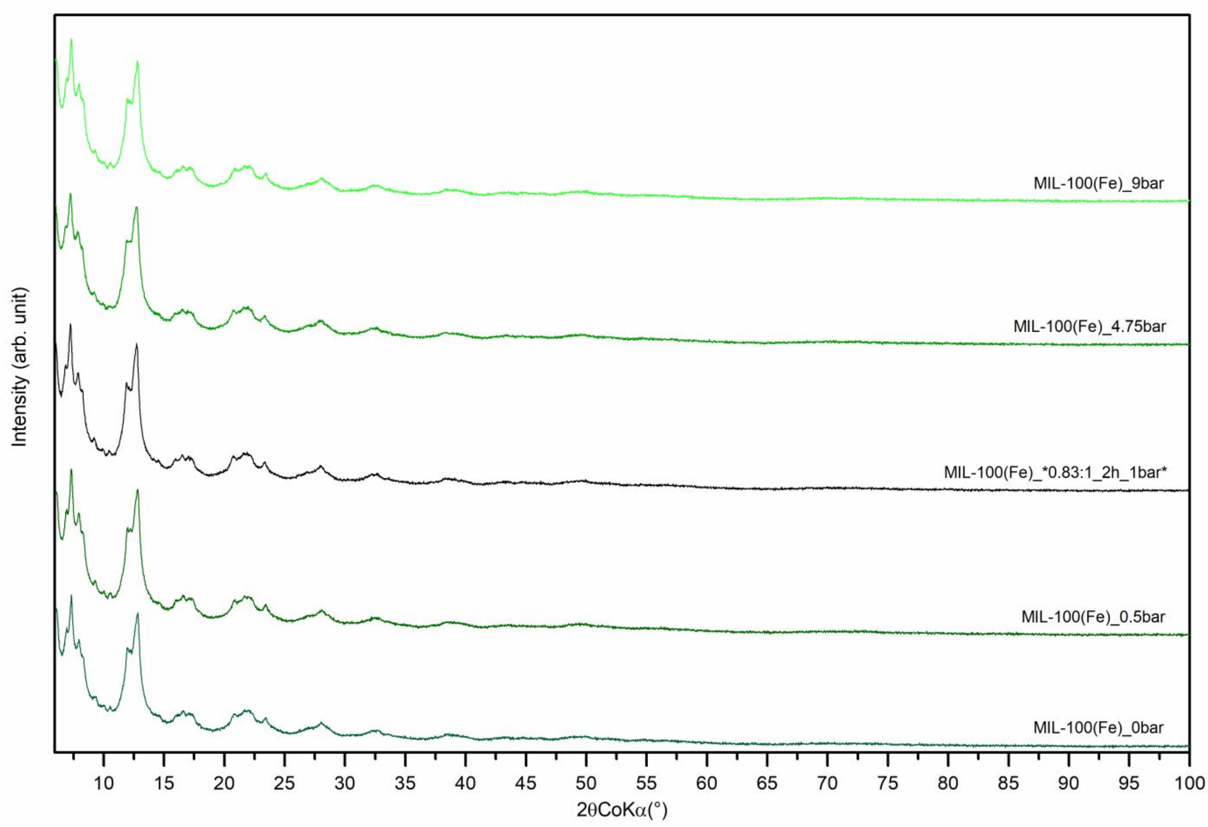

Figure S4. The evolution of the sample crystallinity through the initial pressure series.

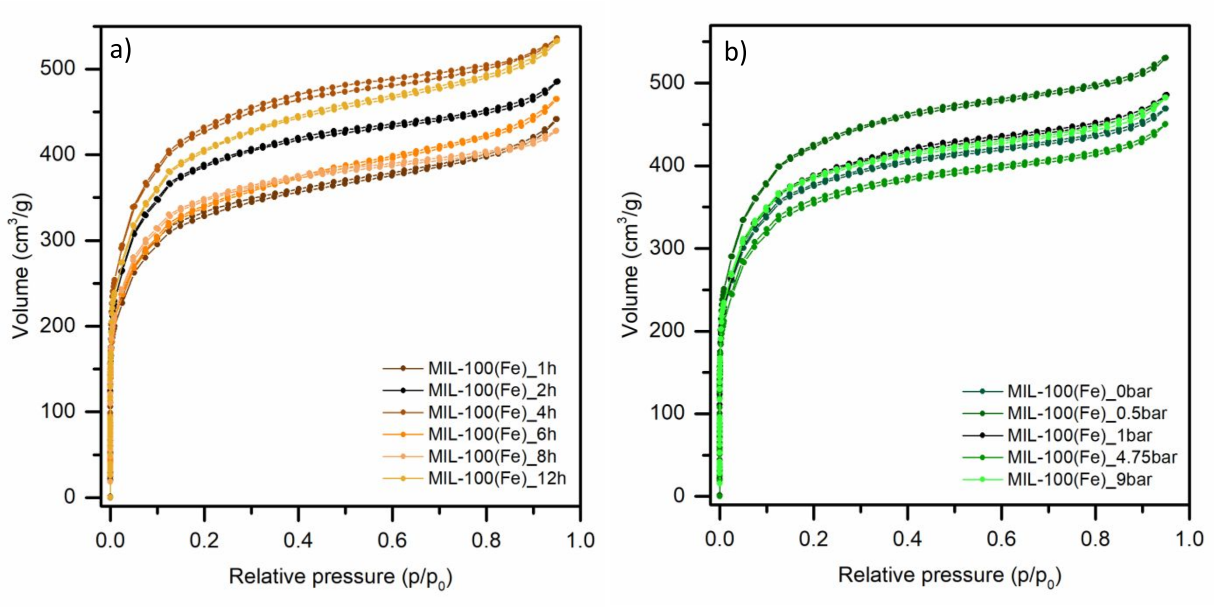

Figure S5. N_2 adsorption-desorption isotherms of the a) reaction time series and b) initial pressure series.s

Table S1. BET surface areas of all measured samples.

Sample	BET area [m^2/g]
**Reaction time series:**	
MIL-100(Fe)_1h	1247
MIL-100(Fe)_*0.83:1_2h_1bar*	1466
MIL-100(Fe)_4h	1615
MIL-100(Fe)_6h	1266
MIL-100(Fe)_8h	1311
MIL-100(Fe)_12h	1518
**Initial pressure series:**	
MIL-100(Fe)_0bar	1419
MIL-100(Fe)_0.5bar	1592
MIL-100(Fe)_*0.83:1_2h_1bar*	1466
MIL-100(Fe)_4.75bar	1347
MIL-100(Fe)_9bar	1419
**Precursor concentration series:**	
MIL-100(Fe)_*0.83:1_2h_1bar*	1466
MIL-100(Fe)_1.66:1	1620
MIL-100(Fe)_3.33:1	1775
MIL-100(Fe)_6.66:1	1607

Table S2. CO_2 sorption of the MIL-100(Fe)_*0.83:1_2h_1bar* measured at different temperatures and across five cycles at 298 K.

**Sample** MIL-100(Fe)_*0.83:1_2h_1bar*	**CO_2 sorption [mmol/g]**	**Sample** MIL-100(Fe)_*0.83:1_2h_1bar*	**CO_2 sorption [mmol/g]**
278 K	2.22	Cycle_1	1.56
288 K	1.97	Cycle_2	1.47
298 K	1.56	Cycle_3	1.54
308 K	1.34	Cycle_4	1.50
		Cycle_5	1.54

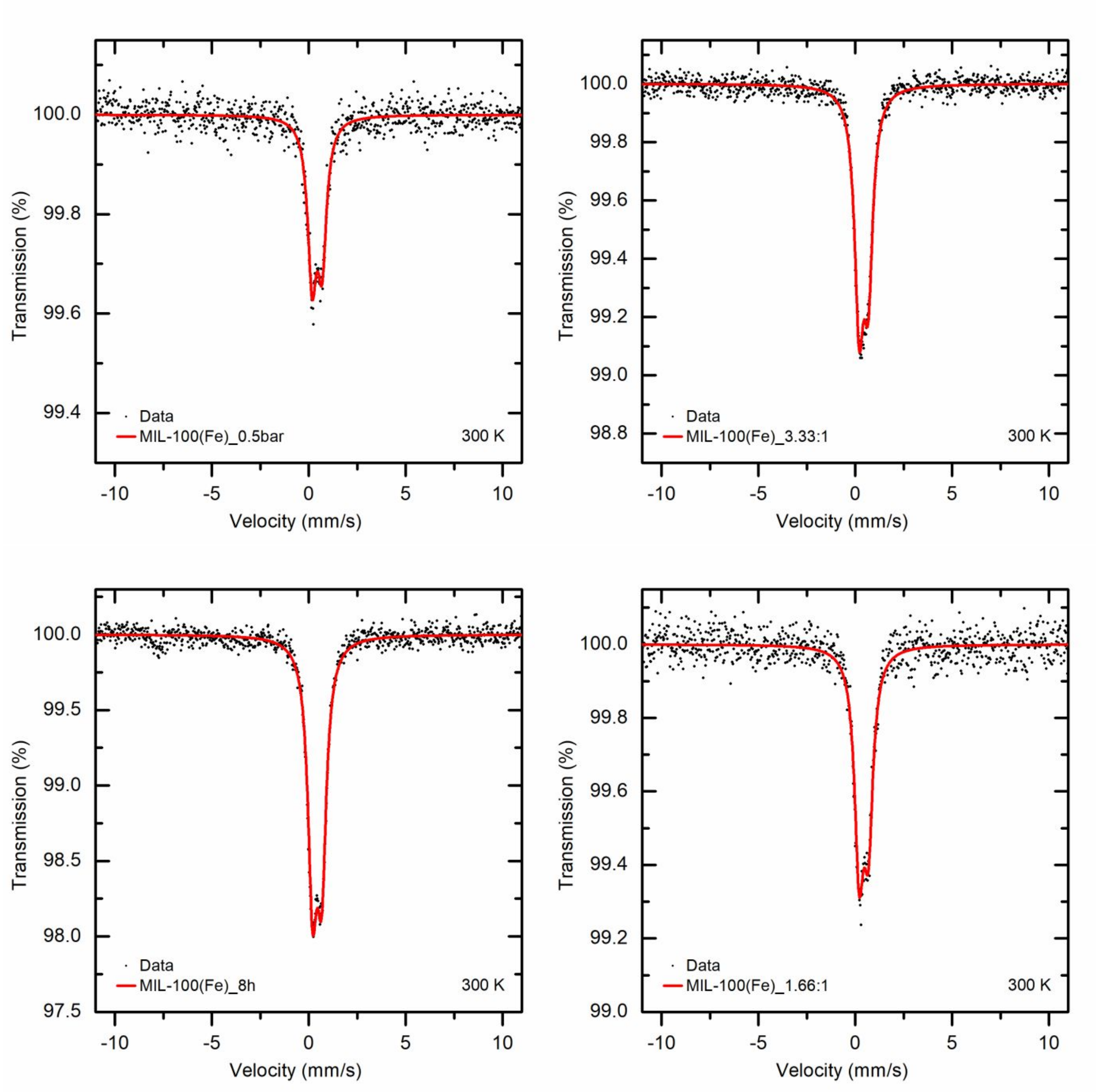

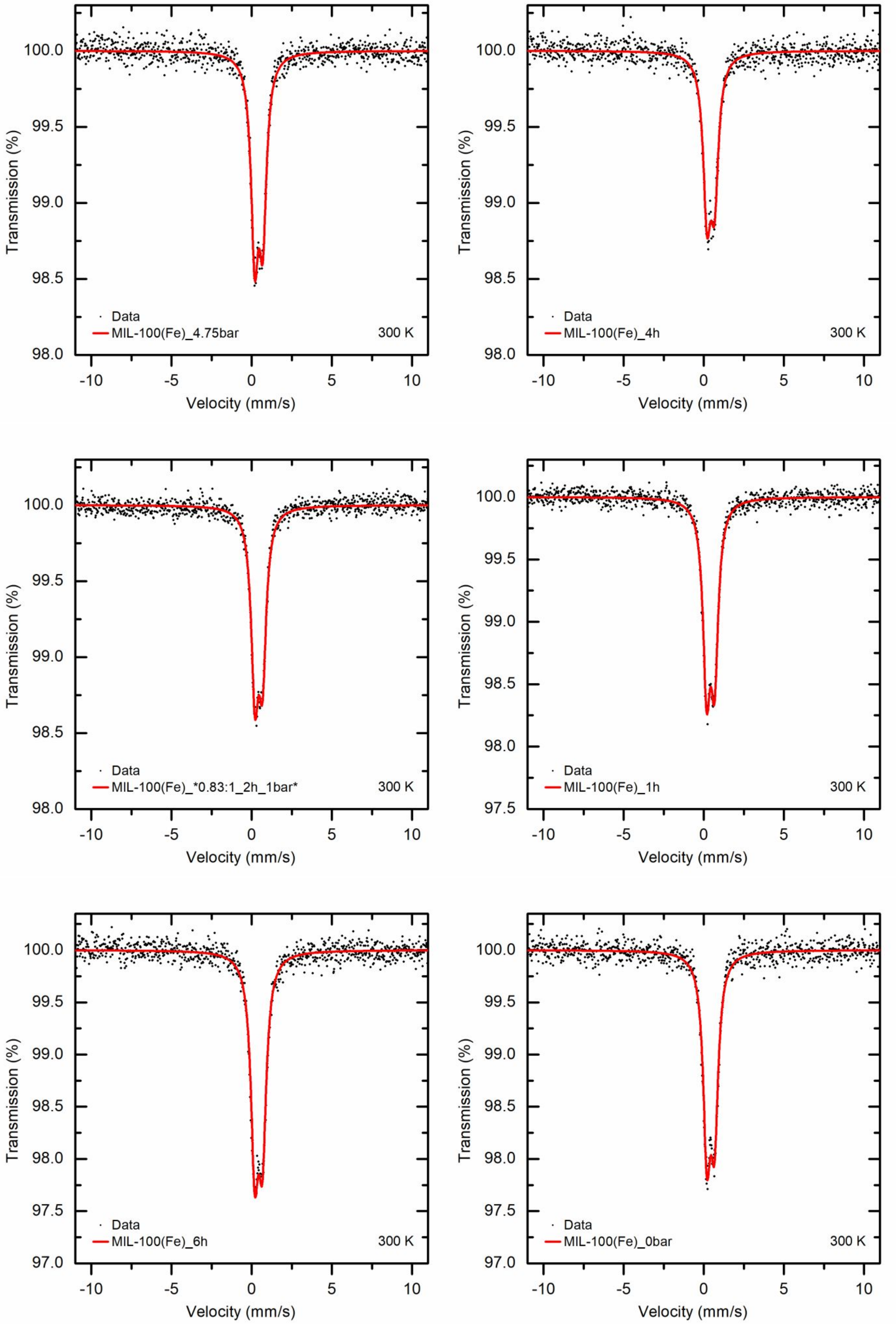
Transmission (%)
Velocity (mm/s)
Data
MIL-100(Fe)_4.75bar
300 K
MIL-100(Fe)_4h
MIL-100(Fe)_*0.83:1_2h_1bar*
MIL-100(Fe)_1h
MIL-100(Fe)_6h
MIL-100(Fe)_0bar

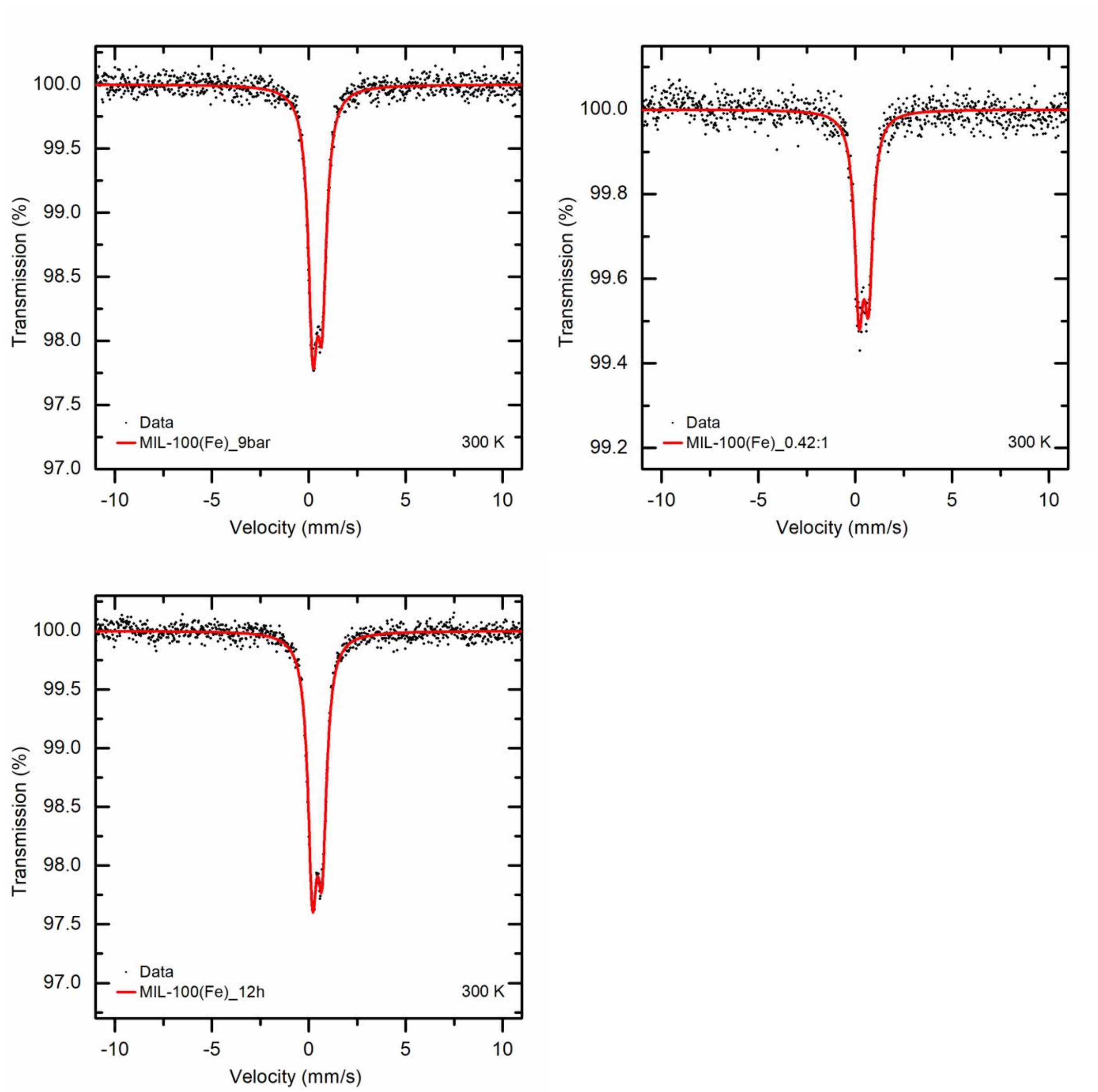

Figure S6. Mössbauer spectra of all prepared samples measured at room temperature. Hyperfine parameters are provided in Tab. S3.

Table S3. Hyperfine parameters of the studied samples evaluated from the Mössbauer spectra, including the amplitudes of spectral lines and their ratio.

**Sample**	**δ[a] ± 0.01 (mm/s)**	**ΔE_Q[b] ± 0.01 (mm/s)**	**Γ[c] ± 0.01 (mm/s)**	**L_1[d] (counts)**	**L_2[e] (counts)**	**L_1/L_2**
**MIL-100(Fe)_ *0.83:1_2h_1bar***	0.43	0.47	0.56	64803	57111	1.134685
**MIL-100(Fe)_1h**	0.42	0.49	0.55	54981	51086	1.076244
**MIL-100(Fe)_4h**	0.43	0.47	0.58	29504	26126	1.129297
**MIL-100(Fe)_6h**	0.43	0.48	0.58	32768	30070	1.089724
**MIL-100(Fe)_8h**	0.43	0.47	0.58	75989	69276	1.096902
**MIL-100(Fe)_12h**	0.44	0.49	0.57	63492	55889	1.136038
**MIL-100(Fe)_0bar**	0.43	0.47	0.56	29845	26759	1.115326
**MIL-100(Fe)_0.5bar**	0.43	0.51	0.56	48472	42496	1.140625
**MIL-100(Fe)_4.75bar**	0.44	0.50	0.56	39418	34884	1.129974
**MIL-100(Fe)_9bar**	0.44	0.47	0.57	45515	39097	1.164156
**MIL-100(Fe)_3.33:1**	0.43	0.47	0.57	117076	96868	1.208614
**MIL-100(Fe)_1.66:1**	0.44	0.48	0.58	43673	36479	1.197209
**MIL-100(Fe)_0.42:1**	0.43	0.49	0.55	46671	42560	1.096593

[a] isomer shift, [b] quadrupole splitting, [c] linewidth, [d] amplitude of the left and [e] right spectral line.

Table S4. Hyperfine parameters of the MIL-100(Fe)_6.66:1 sample evaluated from the Mössbauer spectra, measured at room temperature and at 5K.

Sample	Component	δ[a] ± 0.02 (mm/s)	ΔE_Q[b] ± 0.02 (mm/s)	B_{hf}[c] ± 0.1 (mm/s)	Γ[d] ± 0.02 (mm/s)	RA[e] ± 1 (%)
300 K	Doublet	0.42*	0.50*	-	0.56*	85
	Singlet	0.38	-	-	0.37	15#
5 K	Doublet	0.53	0.46	-	0.53	92
	Sextet	0.50	-0.21	53.0	0.30	8

[a] isomer shift, [b] quadrupole splitting, [c] hyperfine magnetic field, [d] linewidth, [e] relative spectral area. *The marked values were fixed during the evaluation. #The percentage representation of the singlet cannot be determined precisely due to the asymmetry of the MOF doublet spectral lines, which could not be fitted here because of the overlap of spectral components.

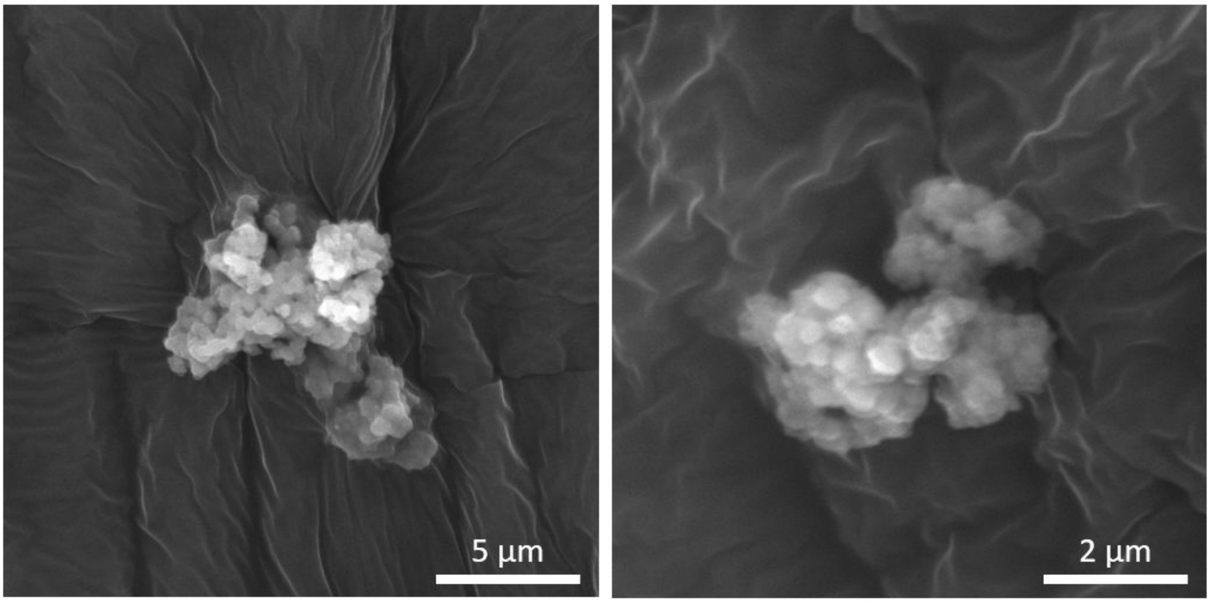

Figure S7. Scanning electron microscope image of MIL-100(Fe)_1h sample.

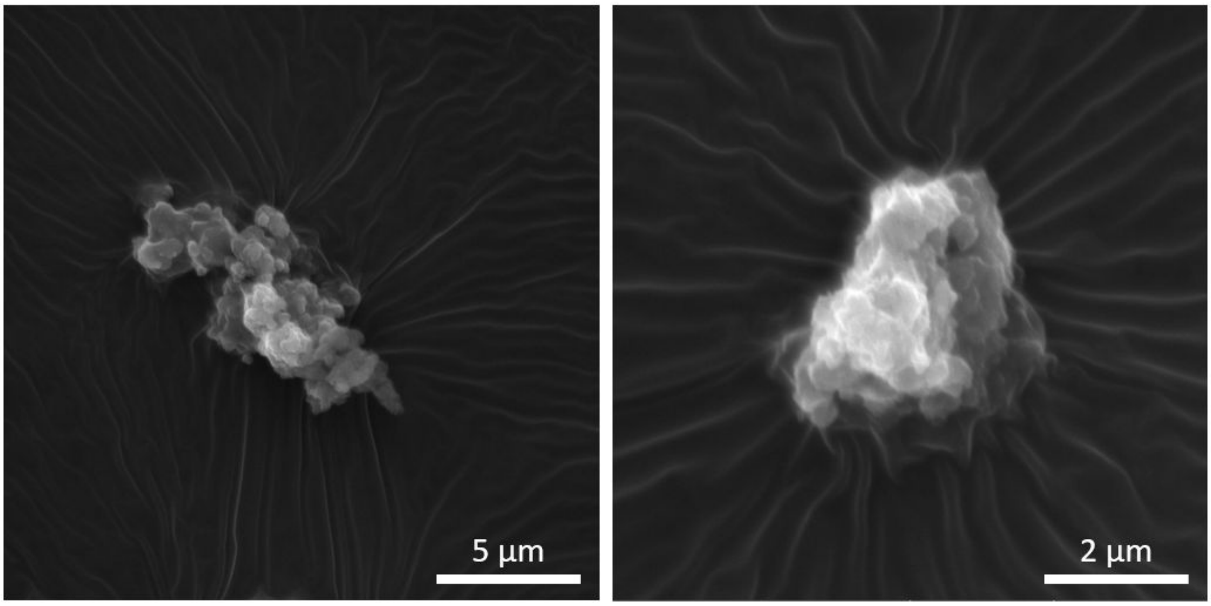

Figure S8. Scanning electron microscope image of MIL-100(Fe)_*0.83:1_2h_1bar* sample.

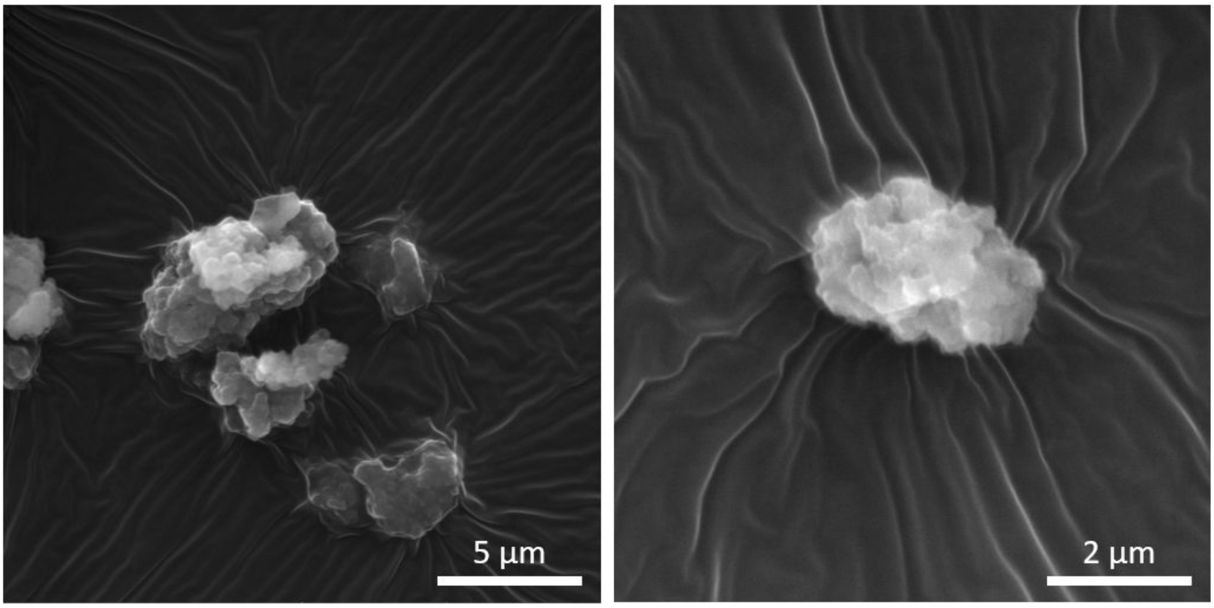

Figure S9. Scanning electron microscope image of MIL-100(Fe)_4h sample.

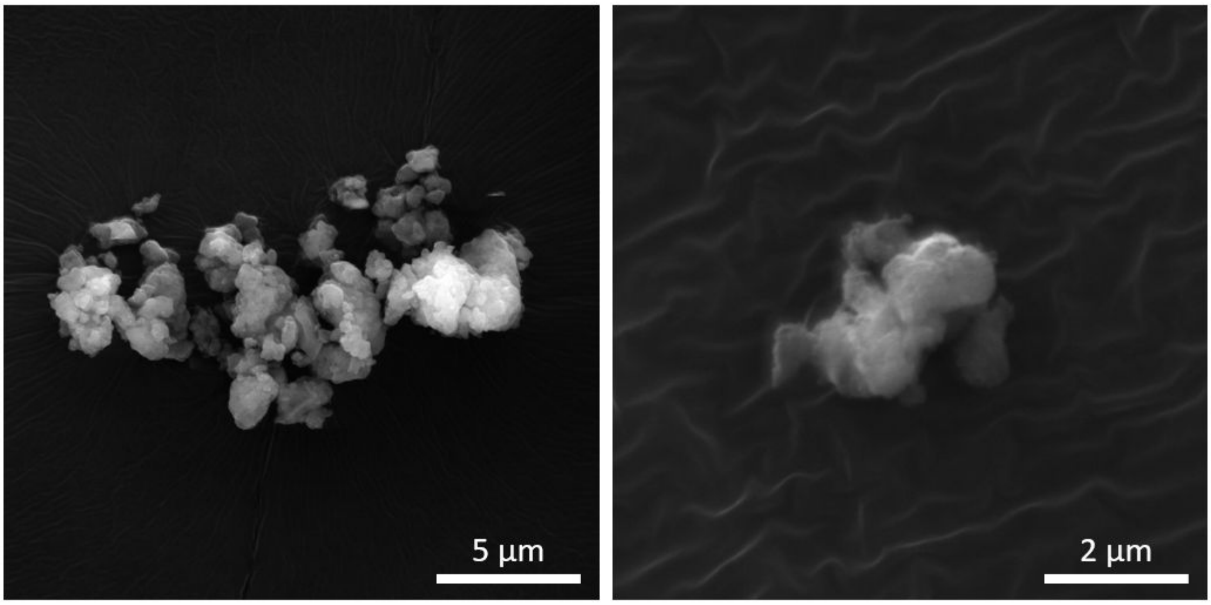

Figure S10. Scanning electron microscope image of MIL-100(Fe)_1.66:1 sample.

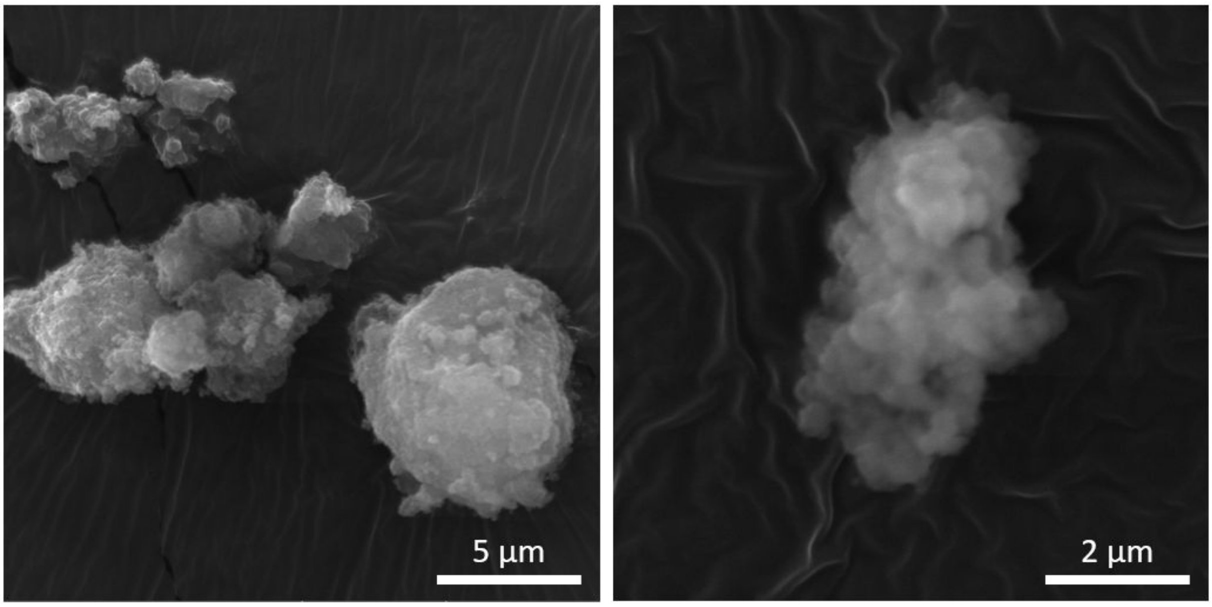

Figure S11. Scanning electron microscope image of MIL-100(Fe)_3.33:1 sample.

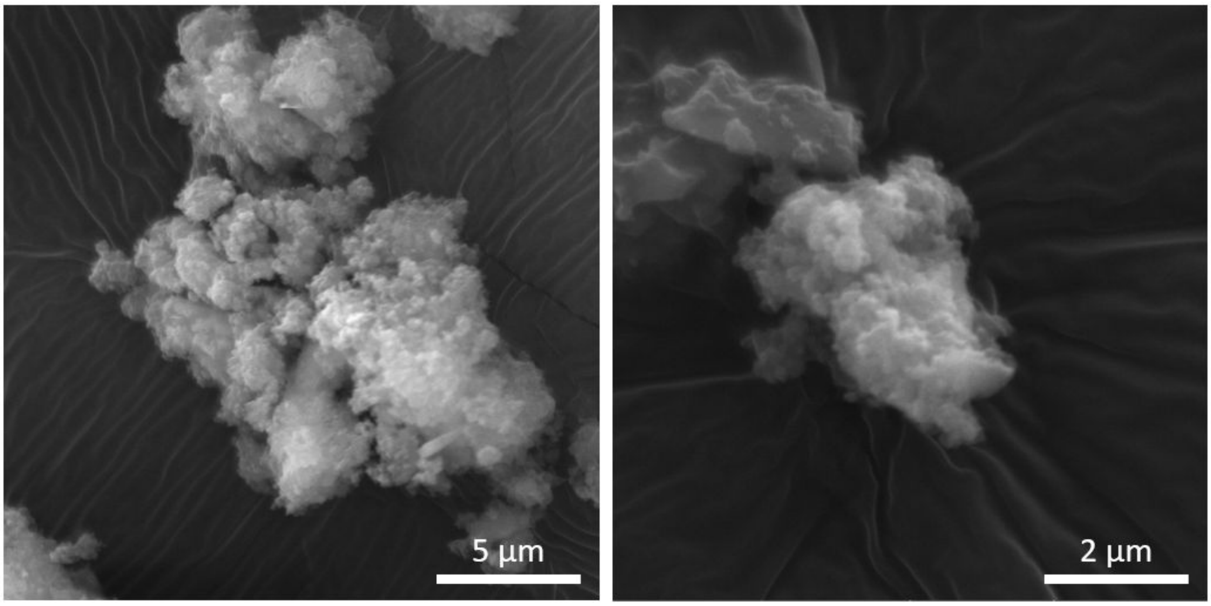

Figure S12. Scanning electron microscope image of MIL-100(Fe)_6.66:1 sample.
